# Ultrafast Solid-Phase Oxidation of Aldehydes to Carboxylic Acids by Atmosphseric Plasma Treatment

**DOI:** 10.1021/acsomega.4c01596

**Published:** 2024-06-06

**Authors:** Bálint Árpád Ádám, Ádám Golcs, Tünde Tóth, Péter Huszthy

**Affiliations:** † Department of Organic Chemistry and Technology, Budapest University of Technology and Economics, Szent Gellért tér 4., H-1111 Budapest, Hungary; ‡ Department of Pharmaceutical Chemistry, Semmelweis University, Hőgyes Endre utca 9., H-1092 Budapest, Hungary; § HUN-REN Centre for Energy Research, Konkoly-Thege Miklós út 29-33., H-1121 Budapest, Hungary

## Abstract

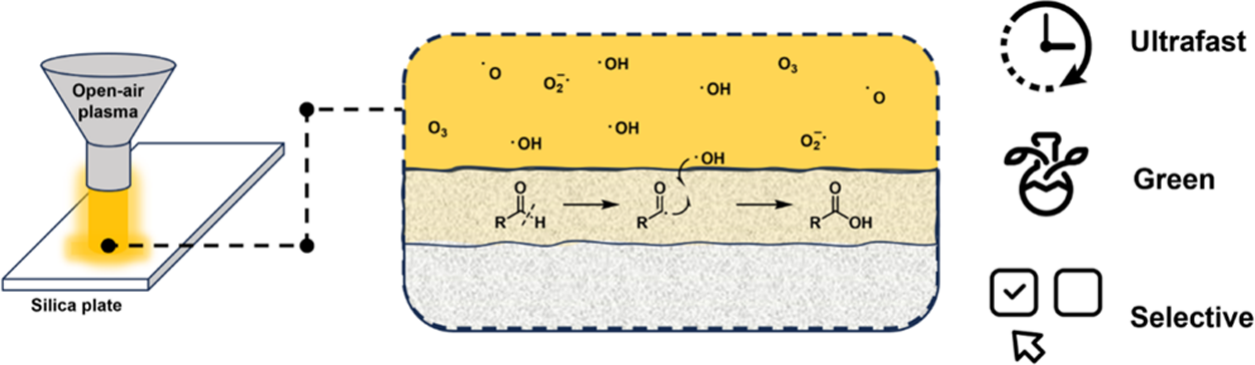

Although atmospheric plasma treatment is an industrially widespread, scalable, and environmentally friendly method, it has been generally used for surface modification, decontamination, or sterilization. In this paper, a novel, sustainable, green, and ultrafast oxidation method is described for aldehydes on a preparative thin-layer chromatographic plate as a solid support. The plasma treatment has proven to be suitable for producing the corresponding carboxylic acids by using only air as a reactant source under mild reaction conditions, while the isolation of the products is also directly integrated into the oxidation process. Extensibility to other reaction types is not explored yet, but we are sure that this novel synthesis conception carries a lot of possibilities.

## 1 Introduction

Plasma treatment has long been known as one of the most widely used and industrially adapted physical type surface modification techniques for polymers.^1^ This modification carries multiple functions like etching, removing surface contaminants, triggering oxidation processes, cross-linking, and increasing the polarity and wettability of the surface of a polymer for better adhesion or further functionalization.^2,3^ From an industrial adaptability point of view, the atmospheric plasma is of particular interest, as it is a much cheaper, faster, and easier-to-implement alternative to low-pressure or argon-based ones.^4,5^ The functionalization of the polymer surface by atmospheric air plasma typically introduces polar hydroxyl (−OH), carbonyl (>C=O), and carboxyl (−COOH) groups on an originally apolar material surface besides the chemical interconversion of oxidizable functional groups (*e.g*., −CN).^6,7^ Despite the widespread use of plasma processes, its mechanism of action is not completely explored^8^ and the chemical conversions are generalized by reactions with hydroxyl radicals (^•^OH) or single oxygen radical (˙O).^6−9^ Consequently, the focus of applicability to polymer treatment comes from the lack of selectivity and controllability. The number and position of the introduced functional groups vary even by infinitesimal changes, while the functionalization is usually accompanied by chain breaking, partial decomposition, or conformational change.^8,10^ That is why the chemical application of plasma as a source of active oxidizing species is generally coupled with catalytic processes to specify structural conversions.^11−14^ Accordingly, both biological and chemical decontaminations and remediations are also among the well-exploitable possibilities.^15−21^

Despite the extensive application of atmospheric plasma treatment for the former purposes, it has not been fully exploited in organic chemical syntheses. The most well-explored fields of plasma-induced oxidative reactions are wastewater treatment^16,22^ and industrial gas pollutant abatement.^23−25^ The aim of the treatment, in such cases, is to oxidize the organic compounds to smaller, nontoxic molecules,^26^ not the production of valuable chemical materials. Therefore, the selectivity is not emphasized in this type of research. In contrast, oxidation in “constructive” organic syntheses is much less investigated due to the fact that the plasma treatment is a radical, hardly controllable intervention.^27^ In a recent study, researchers successfully produced propylene oxide from propylene gas with high selectivity in the presence of a titanium silicate catalyst using plasma.^28^ However, the use of metal catalysts should be avoided from a green chemistry point of view. Results are available, where hydrocarbons^27^ and glycerol^29^ are transformed into a mixture of alcohols, aldehydes, ketones, and carboxylic acids without any catalysts in lab-scale equipment. In these reactions, the selectivity is lower, requiring extra effort to separate the products. The aim of nowadays’ studies to increase both the selectivity and conversion by reducing the scale of reactions and using micro plasma reactors.^30−32^ However, a mixture of products is typically expected in all plasma-assisted reactions.

Carboxylic acids are important compounds with various applications in pharmaceutics and materials science and also as intermediates with a broad synthetic scope.^33−35^ Although oxidation of aldehydes to carboxylic acids is one of the most universally applied synthetic conversions, which can also take place spontaneously during a long-time storage under air, traditional oxidation methods still suffer from the requirement of hazardous reactants, *e.g*., chromates.^36^ Despite the development of improved metal-catalyzed synthetic methods from the past decade,^37−47^ more sustainable and efficient catalyst ligand- and metal-free strategies for aerobic oxidation are highly desired, especially the ones using only a small amount of solvent to reduce the environmental impact.^48,49^ Apart from the triviality of this chemical reaction, many efforts were made, even very recently, to overcome the drawbacks and to tailor novel methods for today’s requirements. These recent methods are mainly designed alongside sustainability and green chemistry-oriented considerations.^49−55^ However, many of them still suffer from the need of additional catalysts,^49−55^ even if these catalysts are considered environmentally friendly ones.

In our research, we aimed to investigate the applicability of industrially widespread atmospheric plasma treatment in different oxidation reactions. In our experiments, we treated a solid carrier [preparative thin-layer chromatographic (PTLC) plate for model reactions in the present case] by the oxidizing radicals generated *in situ* from air. On the other hand, advanced implementations, *i.e*., flow-through cells, might also carry potential for industrial adaptation in the future.

## 2 Results and Discussion

During the study, the air pressure, applied voltage, and electric current were maintained at a constant value, while the time of the plasma treatment (hereafter, we will call this as “time” in seconds), distance from the plasma head (hereafter, we will call this as “distance” in mm), and thickness of the solid phase (silica layer of the PTLC plate in mm, hereafter called as “layer thickness”) were the 3 factors of the experiments. Studying the effects of the treatment parameters was carried out according to a full-factorial matrix experimental design. The investigated levels of the factors are summarized in Table 1.

**Table 1:** Levels of the Variables in Parameter Optimization for Oxidation by Plasma Treatment

	adjusted levels of factors		
factors	“low” level	“medium” level	“high” level
layer thickness (mm)	0.2	0.5	2.0
distance (mm)	10	20	30
time (s)	3	6	9

**Figure 1:**
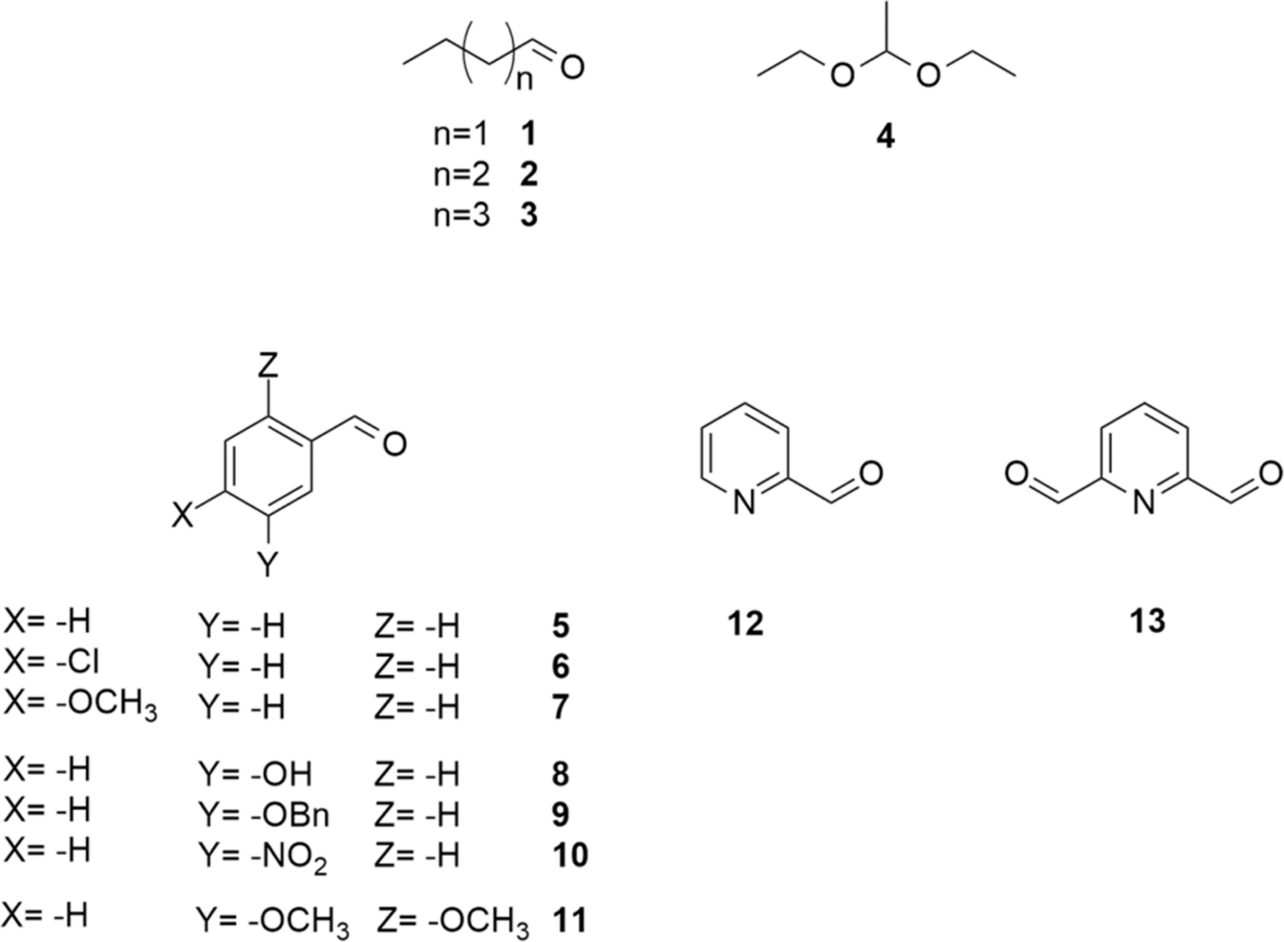
Model compounds for studying the applicability of atmospheric plasma treatment for the oxidation of aldehydes.

We used simple and easily available aldehydes for model compounds. As aliphatic starting materials, the oxidation of butanal (**1**), pentanal (**2**), and hexanal (**3**) was attempted, extended with acetaldehyde diethyl acetal (**4**) as a protected derivative. Among simple aromatic aldehydes, benzaldehyde (**5**), 4-chlorobenzaldehyde (**6**), 4-methoxybenzaldehyde (**7**), 3-hydroxybenzaldehyde (**8**), 3-benzyloxybenzaldehyde (**9**), 3-nitrobenzaldehyde (**10**), and 2,5-dimethoxybenzaldehyde (**11**) were studied, while oxidation of pyridine-2-carbaldehyde (**12**) and 2,6-pyridinedicarbaldehyde (**13**) was also attempted as an example for heteroaromatic derivatives. The applied model compounds are listed in Figure 1.

As preliminary studies, we also attempted the oxidation of the corresponding alcohols, but all of these reactions failed, since no conversion took place. As our research group has long been interested in the chemistry of acridino compounds, several experiments were carried out with the aim of converting variously substituted acridines to the corresponding acridine-9(10H)-ones (oxidation and simultaneous dearomatization). These test reactions resulted in no conversion either (failed test reactions for studying substrate scope and extendibility of the method can be found in the Supporting Information in Figure S1).

The failed tests indicated that the plasma was only able to induce the easiest oxidation processes like the aldehyde–carboxylic acid conversions under the investigated mild conditions. Accordingly, the plasma treatment did not affect the silica support, which proved to be chemically indifferent in the oxidation process. These statements were proved by scanning electron microscopy (SEM), attenuated total reflectance Fourier transform infrared spectroscopy (ATR-FTIR), and contact angle measurements. Moreover, control experiments were also carried out. Further information can be found in the Supporting Information.

It is also important to note that the applied silica gel adsorbent confined the upper limit (relatively harsh conditions) of the parameter setup as intensive exposition to plasma (within 15 mm distance or for more than 10 s) caused the degradation of the solid carrier phase. (Replacing the solid carrier might pave the way for oxidations requiring harsher conditions, but a reduction in selectivity would also be expected in this case.)

The acetal-protected derivative of aldehyde could also not be converted to the corresponding acid, as the protective group was not removed during the plasma treatment. This is favorable as selectivity can be further enhanced by applying protective groups if more than one formyl group is simultaneously present in the molecule.

The results obviously showed that the increased layer thickness had an adverse effect on oxidation; thus, oxidations could only be performed when using 0.2 mm thick plates (this factor was eliminated from the experimental design for further studies). This is not surprising as the reactive radicals can reach the molecules inside the bulk phase of the solid carrier less effectively, while a thin layer has a better permeability for gaseous reagents.

Yields of the oxidation tests as a function of process parameters (distance and time) are shown on the surface diagrams. Based on the calculated statistical characteristics (ANOVA), all of the examined parameters had significant effects on the output. The adequacy of the model was checked by *F*-probe. According to the calculations, we accept the null hypothesis because the probability (*p*) is >0.05 regarding the “lack of fit” factor, *i.e*., the fitted nonlinear model is adequate. All effects of the investigated factors proved to be significant at a significance level of 95%.

Moreover, the adequacy of the model fitted during the experimental design was also examined using a graphical representation of the residues as diagnostics. Information related to the statistical analyses of the experimental data as well as the obtained yields, the investigation of the normality of distribution, constant variance, and independence of the fitted nonlinear model can be found in the Supporting Information.

**Figure 2:**
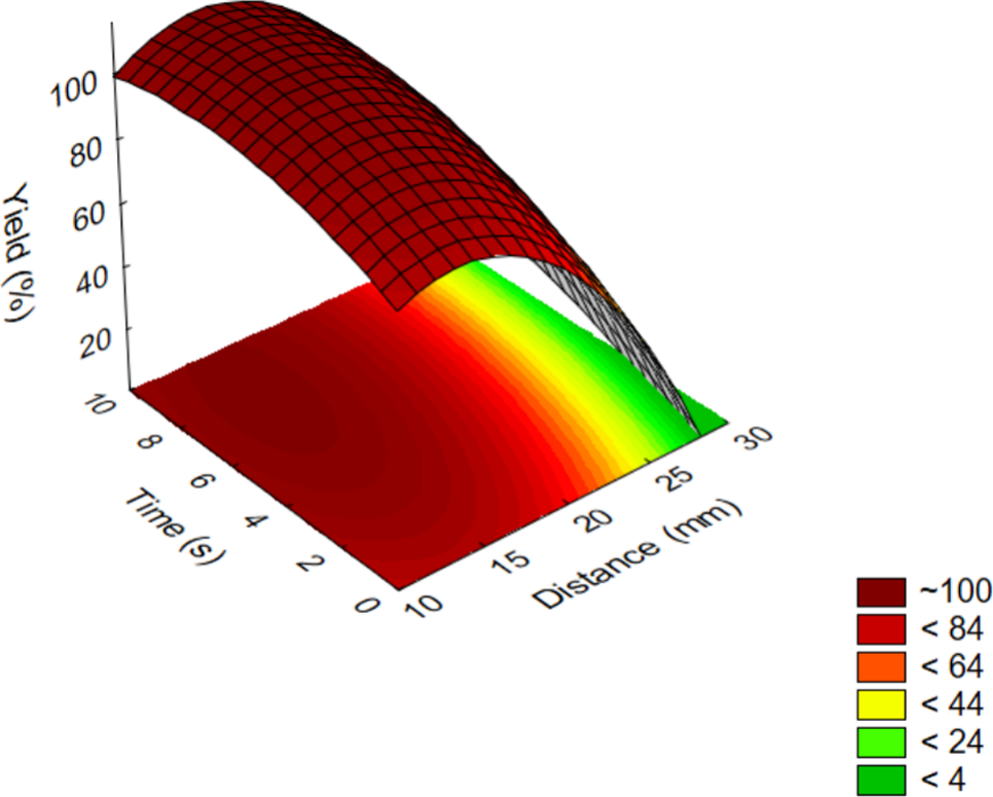
Influence of parameters on the yield of oxidations by plasma treatment for aliphatic model compounds (**1**–**3**; out-of-range values come from an extrapolation based on the measurement data).

The results of the oxidation test for the case of the aliphatic model compounds (**1**–**3**) were summarized and visualized on a three-dimensional (3D) response surface (Figure 2).

The length of the carbon chain of aliphatic derivatives (**1**–**3**) did not influence the efficiency. As it was mentioned above, effective oxidation could only be performed when using 0.2 mm thick plates as solid supports.

Among the other two investigated factors, the effect of treatment time was found to be negligible since almost complete conversions were achieved even within a few seconds. In contrast, the treatment distance had a much stronger effect on the results. Below a distance of 15 mm, the silica support suffered partial decomposition. This adversely affected the removal of the product from the stationary phase during the chromatographic isolation in the final step of the process (see step 4 in Figure 7). However, above this distance, the intensity of the treatment decreased according to the expectations, which resulted in an exponential decrease in the conversion. Above a distance of 25 mm, only a negligible conversion was obtained, and after the procedure, almost the entire amount of the starting material was still present as an aldehyde.

Conversion can be improved by increasing the reaction time; however, according to the previous correlations, it is observed only to a small extent on the examined time scale. In summary, full conversion to the corresponding carboxylic acids could be obtained without the formation of any undesired byproducts using the optimal conditions, which are 8 s of reaction time and 15 mm of distance.

**Figure 3:**
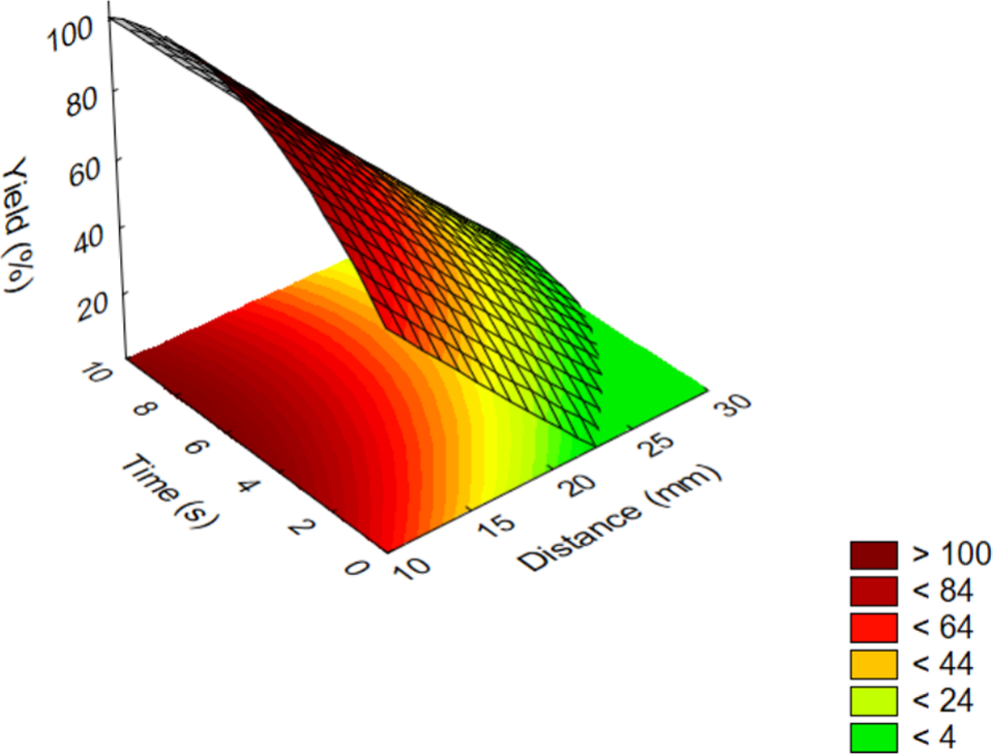
Influence of parameters on the yield of oxidations by plasma treatment for aromatic model compounds (**5**–**11**; out-of-range values come from an extrapolation based on the measurement data).

Figure 3 shows the results obtained for aromatic model compounds (**5**–**11**).

The expected substituent effects (due to the presence of electron-donating and -withdrawing functional groups at *para*-, *meta*-, or *ortho*-position of the aromatic ring) could not reflect in the results; thus, the yields for the aromatic model compounds (**5**–**11**) could be presented in one diagram. Probably, these effects are present but are too weak to manifest near the obtained deviations (yields within ±5.0%) of the parallel results.

In contrast, the relative reactivity between aliphatic (**1**–**3**) and aromatic aldehydes (**5**–**11**) was revealed according to the expectations and showed a higher tendency of the former to oxidation. (In the case of aromatic aldehydes, the partial positive charge of the formyl-*C* is stabilized by the delocalized π-electron system.) Similar to the aliphatic derivatives (**1**–**3**), increasing the distance and the time reversely affected the conversion, causing a gradient change in output even upon a slight altering of the parameter values. On the other hand, the time factor had a greater effect on yields in this case. It can be seen that an almost full conversion could be reached once exceeding 6 s of treatment, while order of minutes was needed to gain carboxylic acids with a high yield when treating from a distance above 25 mm. The optimum was at 7 s and from 15 mm distance. Under harsher conditions, only the previously described adverse effects are expected to take place. Similarly, longer time treatment from the optimal distance can also result in the decomposition of the silica support, thus keeping the optimal conditions is strongly encouraged. It can be concluded that within the studied parameter range, the process is more sensitive to changing the distance, so the choice of this parameter is more critical. In contrast, slightly exceeding the suggested reaction time is allowed to maintain the results close to the optimum.

It can also be seen that the mathematical model of the parameter dependence differs significantly for each compound group (*e.g*., almost linear distance dependence in Figure 3, while exponential dependence on the same parameter in Figure 2). This is related to the fact that in such heterogeneous reactions, which are presumably partly radical and gas-diffusion-controlled, the reactivity of the different compound types is not only defined by the oxidation tendency from a chemical context. The polar surface area, the characteristics of the molecular distribution on the porous solid phase, and the supporting or even inhibiting effects of the microenvironment of the heterogeneous reactions can all play a critical role. As a result, the combination of these effects is manifested in the shape of the surface diagrams.

**Figure 4:**
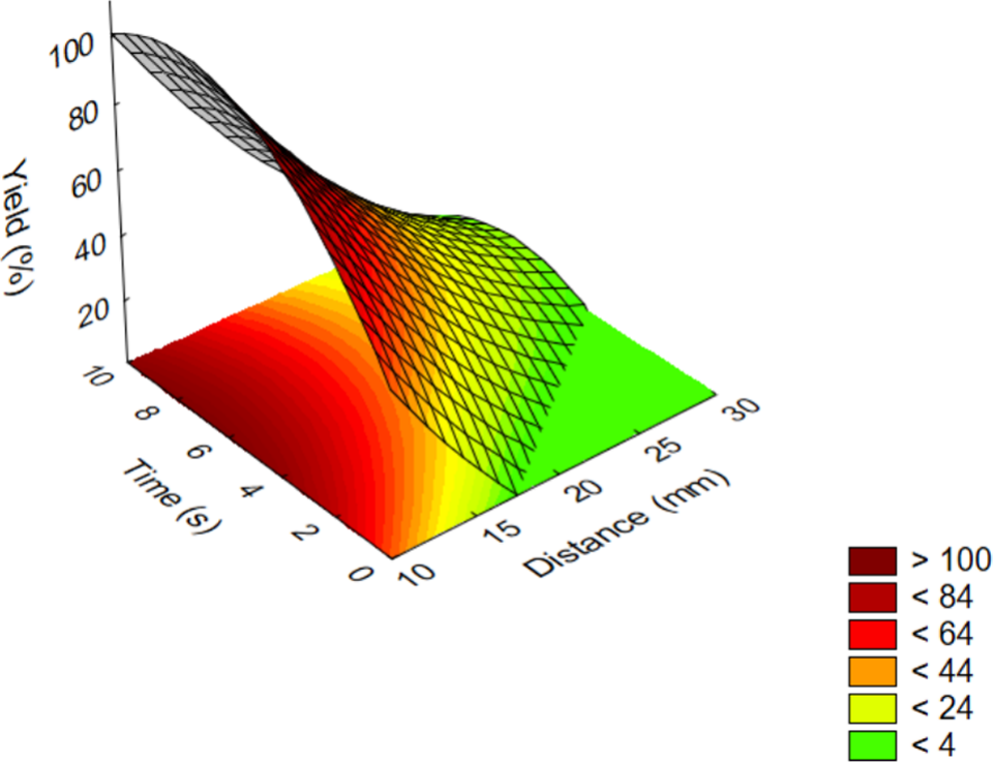
Influence of parameters on the yield of oxidations by plasma treatment for heteroaromatic model compounds (**12**,**13**; out-of-range values come from an extrapolation based on the measurement data).

Figure 4 represents the results for the heteroaromatic model compounds (**12**,**13**).

The small difference in the overall reactivities of aromatic (**5**–**11**) and heteroaromatic (**12**,**13**) derivatives did not prove to be statistically significant when applying the optimal conditions. On the other hand, the nature of the parameter dependence showed a significant difference in the studied condition range. The time optimum was similarly 7 s, but the reaction was more sensitive to the kinetics, since the conversion changed to a greater extent depending on the duration of the treatment in this case. Moreover, a slight decrease in yields was also observed even within 10 s of reaction time. On this studied time scale, it is recommended to use a distance within 20 mm to ensure a favorable output. In the case of the heteroaromatic model compounds (**12**,**13**), in addition to time, the effect of distance was also more significant compared to the previous compound groups. The yield of the oxidation increased gradually as a function of the distance.

It is interesting that under optimized conditions, only one of the formyl groups of the binary aldehyde (**13**) was oxidized and almost the same yields were obtained as in the case of 2-pyridinecarboxaldehyde (**12**). It is obvious that the active oxidizing radicals are present only for a short time in the gas phase during the reaction. The oxidation of both aldehyde units would require longer treatment. Unfortunately, this was not possible due to the observed partial degradation of the applied silica support. This limitation might be eliminated by replacing the solid support with more advanced carriers in the future, which would allow the application of harsher reaction conditions. Probably, changes in the porosity of the support layer can also enhance the effectivity of the heterogeneous reaction by providing a better phase contact. In summary, we can state that the optimal conditions were determined within the investigated condition range for all types of model compounds. It is important to note that the applied parameter window was specified for the instrument and its basic parameter settings, *i.e*., the pressure of the compressed air source, voltage, electric current, etc. On the other hand, it was clearly demonstrated that the plasma treatment was successfully applicable for the desired purpose and suitable parameter optimization enabled an outstanding performance among literature alternatives of the presented oxidation reactions.

**Figure 5:**
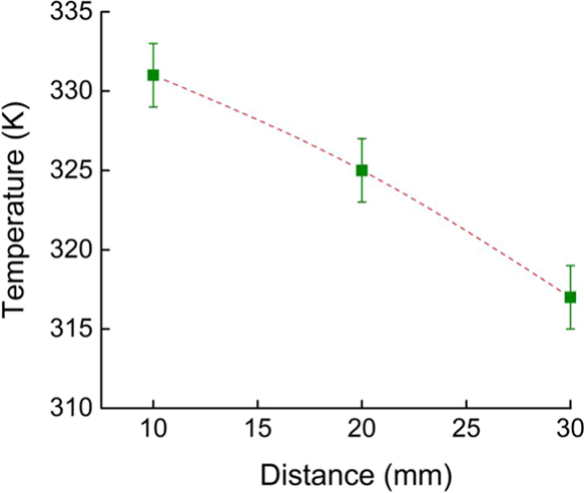
Temperature of the silica surface during the plasma treatment as a function of the distance from the plasma head.

It is also worth mentioning that the parameters cannot be considered fully independent in the present study. Naturally, a smaller distance also means a higher reaction temperature, but the temperature range is relatively narrow. The temperatures, which were measured on the surface of the solid support during the plasma treatment, are shown in Figure 5.

The measured temperatures can be considered mild reaction conditions. However, it is clear that the difference of temperatures as an “embedded parameter” also has a significant role in influencing the rate of the reactions. Thus, in the case of the present discussion, the distance parameter has to be considered as a combined factor, including the effects of the temperature of the reaction estimated by the surface temperature.

In general, side reactions were not observed beside the expected oxidations. After the plasma treatment, only the starting aldehyde and the resulting carboxylic acid were present according to chromatographic isolation. That is why the mass balance was only influenced by the limitations of the recovery of the materials from the solid support, which was above 80% in all of the cases. It is also important to mention that the support was coated by a normal phase stationary phase; thus, the starting aldehydes (more apolar components) showed a higher efficiency in recovery than the resulting carboxylic acids as products. This results in the underestimation of the selectivity for carboxylic acids, as most of the material loss affects the isolated yields of the products.

Although the low number of model compounds does not enable one to make a consequence regarding extendibility in applications, the promising effectivity and favorable properties clearly demonstrated the relevance of the proposed novel oxidation method for considering it among state-of-the-art synthesis alternatives.

## 3 Materials and Methods

Starting materials and reagents were purchased from Sigma-Aldrich (owned by Merck, Darmstadt, Germany) and used without purification. Both for solid carrier support in the oxidation process and for isolation of the reaction products, PTLC Silica Gel 60 F_254_ (Merck, Darmstadt, Germany) plates with different layer thicknesses (2.0 or 0.5 or 0.2 mm) were used [particle size 10–12 μm (d50 laser diffraction, size distribution); pore size 60 Å medium pore diameter; specific surface area (according to Brunauer–Emmett–Teller (BET); 5-Pt. measurement) 480–540 m^2^/g; pore volume (N_2_ isotherm) 0.74–0.84 mL/g; deviation of layer thickness per plate ≤35 μm]. All reactions were monitored by TLC and visualized by a ultraviolet (UV) lamp or by using 2,4-dinitrophenylhydrazine (in sulfuric acid/water/ethanol solution) and bromocresol green (in ethanol solution) for developing stains of the corresponding aldehydes and carboxylic acids, respectively. Ratios of solvents for the eluents are given in volumes (mL/mL). Evaporation was carried out under reduced pressure, unless otherwise stated.

**Figure 6:**
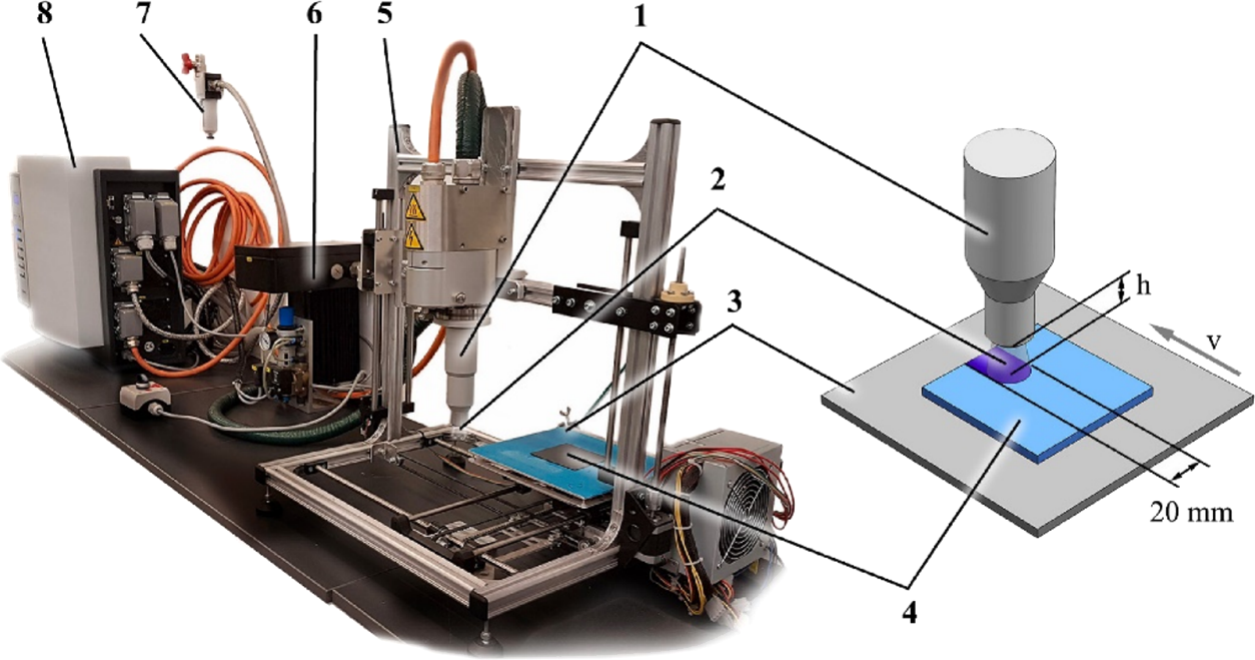
Plasma treatment setup: 1—plasma head, 2—plasma, 3—support plate, 4—PTLC plate (*h*: distance from the plasma head to the surface of the treated PTLC plate, *v*: the linear speed of the support plate), 5—machine moving frame, 6—pressure regulator, 7—compressed air, 8—plasma generator. Adapted with permission from ref (7). Copyright 2022 Elsevier.

The plasma treatment was performed with an FG 5001 plasma generator (Plasmatreat GmbH, Steinhagen, Germany). The plasma was generated from compressed air. The pressure was set by using a gas reductor equipped with a manometer. The compressed air with reduced pressure was introduced into a Plasmatreat RD1004 rotating plasma head. The parameters affecting the plasma properties (voltage, amperage, etc.) were set on the digital control unit. The plasma head was mounted on a frame. The PTLC plates were fixed on a support plate, while the movement of the support plate was controlled by a computer. The instrumentation is shown in Figure 6 (adapted from ref (7)).

Plasma treatment was carried out using an air pressure of 3.5 bar, a voltage of 280 V, and an electric current of 17.5 A. The central 20 mm wide band of the PTLC plate was treated by moving the support plate under the plasma head with different linear speeds, which define the time of the treatments (3–9 s for the same surface area). The vertical distance between the plasma head and the moving table varied from 10 to 30 mm.

All of the compounds, which were used in this study, have already been characterized; thus, the structures of the isolated compounds were checked by comparison with literature data based on TLC and ^1^H NMR measurements (for aliphatic model compounds, see refs (56−59); for aromatic model compounds, see ref (60); for heteroaromatic model compounds, see refs (61,62)). ^1^H NMR (300 MHz) spectra were recorded on a Bruker 300 Avance spectrometer (Bruker Corporation, Billerica, MA).

All of the reported data came from the averages of 3 independent experiments. The experiments were carried out at 298 ± 1 K, while the temperature of the silica surface was determined by an OEMTools digital infrared thermometer (OEMTools Co., Easton, MA) immediately after the plasma treatment, in which data can be used for estimating the temperature of the chemical reactions inside the solid-phase microenvironment. The randomization of the experimental design and the statistical evaluation were carried out using STATISTICA 13.4.0.14 (TIBCO Software Inc.) software. During the statistical investigations, a confidence level of 95% was used in all cases. OriginPro 8.6 (OriginLab Corporation) software was used for the graphical interpretation of the results.

**Figure 7:**
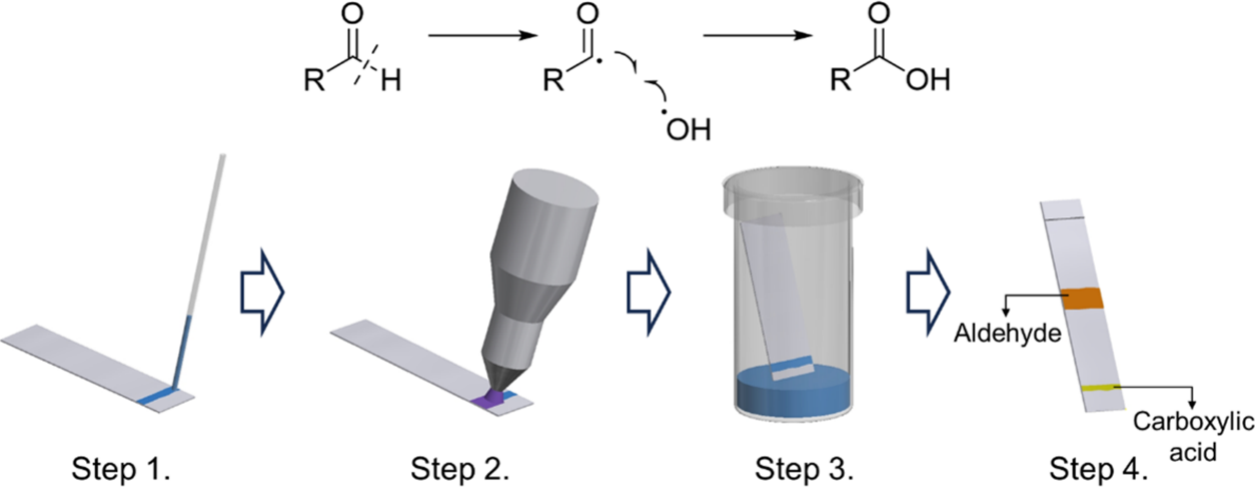
4-Step-protocol of the novel plasma-based oxidation (step 1: spreading the solution of the aldehyde on the silica PTLC plate; step 2: oxidation by atmospheric plasma treatment; step 3: PTLC-based purification of the compounds from the reaction mixture; step 4: isolation of the PTLC fractions by removing the components from the silica gel solid stationary phase).

The procedure of this study is summarized in Figure 7.

In each case, 20 mg of starting aldehydes as a saturated solution in ethanol was evenly spread on the silica support. The starting material was present inside the pores. This approach has several advantages as it provides a better phase contact, which is particularly important in the case of heterogeneous reactions. The distribution on a porous substrate with a large surface area significantly accelerates the reaction and results in a more uniform plasma treatment than in the case of a liquid-plasma heterogeneous system.

Naturally, the plasma contains a mixture of possible active reagents *in situ* generated from the air. All of them can play an active role in oxidizing the aldehydes to carboxylic acids. However, as this conversion also required the addition of an O-atom containing species, the essence of the reaction can be most easily expressed by the reaction of simple ˙O or ˙OH radicals even if other substances (*e.g*., O_3_, NO_*x*_ and its radical and ionic derivatives, peroxyl radical, differently charged O-radical ions, etc.) with oxidizing effects can support the whole process. Reported works generally refer plasma treatment as a radical process.^63^ Although the reaction network can be quite diverse, the following radical steps are probably the most typical ones: 1. initialization through formyl-*H*-abstraction; 2. formation of alkyl and hydroxyl radicals; 3. recombination of ˙OH radicals with alkyl radicals to form carboxylic acids. (The alkyl radical can also react, for example, with the abundant molecular oxygen, while forming a peroxyl radical, which is unstable and immediately undergoes disproportionation.)

The possible effects of the treated silica support layer on the oxidation process were excluded by SEM, ATR-FTIR, and contact angle measurements besides further control experiments. Surface sensitive secondary SEM electron images were recorded by using a LEO 1540XB system (Carl Zeiss AG, Oberkochen, Germany) operated with a system vacuum of 1.0 × 10^–6^ mbar. A minimized acceleration voltage of 1 kV and a low beam current of approximately 40 pA was set to avoid the charging of the analyzed structures considering the low electrical conductivity of the polymer layer. Infrared spectra were recorded on a PerkinElmer Spectrum Two FTIR (PerkinElmer, Inc., Waltham, MA) with a universal ATR accessory head. Contact angle measurements were made by a smartphone camera and software was used to determine the contact angles. A 7.5 μL water droplet was set in a chamber in front of the camera. The whole process was filmed, and the contact angle was measured just before the droplet started to leak into the porous silica. The measurements were made at 25 °C and a humidity of 60%.

After the plasma treatment of the corresponding area of the silica layer, the PTLC plate was immersed in a solution mixture of dichloromethane and ethanol (10:1) to isolate the compounds in the reaction mixture. Ethanol was used for removing the isolated fractions from the silica layer of the PTLC plate. After the mixture was stirred and vacuum-filtered of the silica adsorbent, the filtrate was evaporated to provide the isolated components. The yields were directly determined by weight measurements by using a Mettler Toledo XS105 microanalytical balance (±0.1 mg precision; Mettler Toledo) after evaporation to a constant weight. The aldehydes had a chromatographic retention factor (*R*_f_) of 0.2–0.7, while the corresponding carboxylic acids remained at the *R*_f_ = 0.0–0.1 starting range during the PTLC-based isolation in all cases. The loss of mass compared to the initial amount of the starting aldehyde was below 20 w/w%.

## 4 Conclusions and Perspectives

We have shown by the examples of some aliphatic and aromatic aldehydes that under optimized conditions, the treatment with atmospheric plasma for some seconds is enough to effectively convert aldehydes to the corresponding carboxylic acids even in the solid phase. Under the reported conditions, the reaction was selective for aldehydes. The main advantages of the method are as follows:

1. Unique fastness
2. Needless of solvents for conversion
3. Sustainability and low environmental impact (reactive species generated *in situ* from air)

The model reactions were carried out on a silica-gel-coated thin-layer chromatographic plate, which can be replaced by more advanced solid carriers in the future. Combination with solid-phase catalytic surfaces might broaden the applicability scope of the proposed method. Furthermore, the described concept and the obtained reaction times anticipate the relevance of flow-through implementations in further developments. This reported work provides a novel approach for investigation on plasma chemistry, and we believe that further efforts will enable the successful introduction of this promising tool in various types of organic syntheses.

## Supporting Information Available

Failed test reactions; determined yields in the experimental design; validation of the experimental design; effects of plasma treatment on the silica support (PDF)

## References

[ref1] Hegemann D.; Brunner H.; Oehr C. Plasma treatment of polymers for surface and adhesion improvement. Nucl. Instrum. Methods Phys. Res., Sect. B 2003, 208, 281–286. 10.1016/S0168-583X(03)00644-X.

[ref2] Thomas S.; Mozetic M.; Cvelbar U.; Spatenka P.; Praveen K. M. Non-thermal Plasma Technology for Polymeric Materials: Applications in Composites, Nanostructured Materials, and Biomedical Fields, 1st ed.; Elsevier: Amsterdam, Netherlands, 2019.

[ref3] Erden S.; Ho K. K.; Lamoriniere S.; Lee A. F.; Yildiz H.; Bismarck A. Continuous atmospheric plasma oxidation of carbon fibres: influence on the fibre surface and bulk properties and adhesion to polyamide 12. Plasma Chem. Plasma Process. 2010, 30, 471–487. 10.1007/s11090-010-9227-6.

[ref4] Bárdos L.; Baránková H. Cold atmospheric plasma: Sources, processes, and applications. Thin Solid Films 2010, 518 (23), 6705–6713. 10.1016/j.tsf.2010.07.044.

[ref5] Xiao J.; Zhang X.; Zhao Z.; Liu J.; Chen Q.; Wang X. Rapid and continuous atmospheric plasma surface modification of PAN-based carbon fibers. ACS Omega 2022, 7 (13), 10963–10969. 10.1021/acsomega.1c06818. 35415352 PMC8991902

[ref6] Abenojar J.; Torregrosa-Coque R.; Martínez M. A.; Martín-Martínez J. M. Surface modifications of polycarbonate (PC) and acrylonitrile butadiene styrene (ABS) copolymer by treatment with atmospheric plasma. Surf. Coat. Technol. 2009, 203 (16), 2173–2180. 10.1016/j.surfcoat.2009.01.037.

[ref7] Boros R.; Tatyana A.; Golcs Á.; Krafcsik O. H.; Kovács J. G. Plasma treatment to improve the adhesion between ABS and PA6 in hybrid structures produced by injection overmolding. Polym. Test. 2022, 106, 107446 10.1016/j.polymertesting.2021.107446.

[ref8] Yusupov M.; Dewaele D.; Attri P.; Khalilov U.; Sobott F.; Bogaerts A. Molecular understanding of the possible mechanisms of oligosaccharide oxidation by cold plasma. Plasma Processes Polym. 2023, 20 (2), 2200137 10.1002/ppap.202200137.

[ref9] Ahmadi M.; Nasri Z.; von Woedtke T.; Wende K. D-glucose oxidation by cold atmospheric plasma-induced reactive species. ACS Omega 2022, 7 (36), 31983–31998. 10.1021/acsomega.2c02965. 36119990 PMC9475618

[ref10] Correia D. M.; Ribeiro C.; Sencadas V.; Botelho G.; Carabineiro S. A. C.; Ribelles J. G.; Lanceros-Méndez S. Influence of oxygen plasma treatment parameters on poly (vinylidene fluoride) electrospun fiber mats wettability. Prog. Org. Coat. 2015, 85, 151–158. 10.1016/j.porgcoat.2015.03.019.

[ref11] Zhu X.; Xiong H.; Liu J.; Gan Y.; Xu Z.; Zhou C.; Wang Y.; Jiang Y.; Tu X. Plasma-enhanced catalytic oxidation of ethylene oxide over Fe–Mn based ternary catalysts. J. Energy Inst. 2022, 103, 138–146. 10.1016/j.joei.2022.06.002.

[ref12] Ma H.; Sharma R. K.; Welzel S.; van de Sanden M. C.; Tsampas M. N.; Schneider W. F. Observation and rationalization of nitrogen oxidation enabled only by coupled plasma and catalyst. Nat. Commun. 2022, 13 (1), 402 10.1038/s41467-021-27912-2. 35058443 PMC8776816

[ref13] Yun J.; Wu L.; Hao Q.; Teng Z.; Gao X.; Dou B.; Bin F. Non-equilibrium plasma enhanced oxygen vacancies of CuO/CeO_2_ nanorod catalysts for toluene oxidation. J. Environ. Chem. Eng. 2022, 10 (3), 107847 10.1016/j.jece.2022.107847.

[ref14] Joshi N.; Loganathan S. In situ modification of CuO–Fe_2_O_3_ by nonthermal plasma: insights into the CO_2_-to-CH_3_OH hydrogenation reaction. ACS Omega 2023, 8 (14), 13410–13420. 10.1021/acsomega.3c00915. 37065016 PMC10099434

[ref15] Farooq M.; Rasheed H.; Rehman N. U. Efficacy of Plasma Enhanced Advanced Oxidation Processes for Decontamination of Water Containing Arsenic. Plasma Chem. Plasma Process. 2023, 44, 269–288. 10.1007/s11090-023-10392-1.

[ref16] Tomei G.; Saleem M.; Ceriani E.; Pinton A.; Marotta E.; Paradisi C. Cold plasma for green advanced reduction/oxidation processes (AROPs) of organic pollutants in water. Chem. - Eur. J. 2023, 29, e202302090 10.1002/chem.202302090. 37621157

[ref17] Foligni R.; Mannozzi C.; Ismaiel L.; Capelli F.; Laurita R.; Tappi S.; Rosa M. D.; Mozzon M. Impact of cold atmospheric plasma (CAP) treatments on the oxidation of pistachio kernel lipids. Foods 2022, 11 (3), 419 10.3390/foods11030419. 35159569 PMC8834114

[ref18] Sanito R. C.; You S. J.; Wang Y. F. Degradation of contaminants in plasma technology: An overview. J. Hazard. Mater. 2022, 424, 127390 10.1016/j.jhazmat.2021.127390. 34879580 PMC8500698

[ref19] Takemura Y.; Umeji S.; Ito K.; Furuya S.; Furuta M. Inactivation treatment of bacterial spores contaminated spices by atmospheric plasma jet. Plasma Med. 2014, 4, 89–100. 10.1615/PlasmaMed.2014011969.

[ref20] Vadikkeettil Y.; Subramaniam Y.; Murugan R.; Ananthapadmanabhan P. V.; Mostaghimi J.; Pershin L.; Batiot-Dupeyrat C.; Kobayashi Y. Plasma assisted decomposition and reforming of greenhouse gases: A review of current status and emerging trends. Renewable Sustainable Energy Rev. 2022, 161, 112343 10.1016/j.rser.2022.112343.

[ref21] Graves D. B. Reactive species from cold atmospheric plasma: Implications for cancer therapy. Plasma Processes Polym. 2014, 11 (12), 1120–1127. 10.1002/ppap.201400068.

[ref22] Khlyustova A.; Sirotkin N. Plasma-assisted oxidation of benzoic acid. Front. Chem. Sci. Eng. 2020, 14 (4), 513–521. 10.1007/s11705-019-1825-0.

[ref23] Jiang L.; Wang P.; Zhang Y.; Yao Z. Plasma-catalytic oxidation of chlorobenzene over Co-Mn/TiO_2_ catalyst in a dielectric barrier discharge reactor with the segmented electrodes. J. Environ. Chem. Eng. 2022, 10 (4), 108021 10.1016/j.jece.2022.108021.

[ref24] Chen G.; Mao M.; Chen L.; Zhang G.; Wang Z.; Liu F.; Ye D.; Wu J. Enhanced plasma-catalytic oxidation of methanol over MOF-derived CeO_2_ catalysts with exposed active sites. J. Environ. Chem. Eng. 2022, 10 (6), 108981 10.1016/j.jece.2022.108981.

[ref25] Marotta E.; Schiorlin M.; Rea M.; Paradisi C. Products and mechanisms of the oxidation of organic compounds in atmospheric air plasmas. J. Phys. D: Appl. Phys. 2010, 43 (12), 124011 10.1088/0022-3727/43/12/124011.

[ref26] Qu M.; Cheng Z.; Sun Z.; Chen D.; Yu J.; Chen J. Non-thermal plasma coupled with catalysis for VOCs abatement: a review. Process Saf. Environ. Prot. 2021, 153, 139–158. 10.1016/j.psep.2021.06.028.

[ref27] Nguyen D. K.; Dimitrakellis P.; Talley M. R.; O’Dea R. M.; Epps T. H.; Watson M. P.; Vlachos D. G. Oxidative functionalization of long-chain liquid alkanes by pulsed plasma discharges at atmospheric pressure. ACS Sustainable Chem. Eng. 2022, 10 (48), 15749–15759. 10.1021/acssuschemeng.2c04269.

[ref28] Lee D.; Chen H.-T.; Linic S. Plasma-induced selective propylene epoxidation using water as the oxygen source. JACS Au 2023, 3 (4), 997–1003. 10.1021/jacsau.3c00030. 37124298 PMC10131193

[ref29] Chida T.; Hiromori K.; Shibasaki-Kitakawa N.; Sasaki S.; Kaneko T.; Takahashi A. Application of nonthermal atmospheric-pressure plasma irradiation as a new method for noncatalytic liquid-phase selective oxidation of polyhydric alcohols. Plasma Processes Polym. 2024, 21, e2300163 10.1002/ppap.202300163.

[ref30] Wengler J.; Ognier S.; Zhang M.; Levernier E.; Guyon C.; Ollivier C.; Fensterbank L.; Tatoulian M. Microfluidic chips for plasma flow chemistry: application to controlled oxidative processes. React. Chem. Eng. 2018, 3 (6), 930–941. 10.1039/C8RE00122G.

[ref31] Cameli F.; Dimitrakellis P.; Vlachos D. G. Direct conversion of ethane to oxygenates, ethylene, and hydrogen in a noncatalytic biphasic plasma microreactor. ACS Sustainable Chem. Eng. 2023, 11 (21), 8003–8008. 10.1021/acssuschemeng.3c01594.

[ref32] Nozaki T.; Hattori A.; Okazaki K. Partial oxidation of methane using a microscale non-equilibrium plasma reactor. Catal. Today 2004, 98 (4), 607–616. 10.1016/j.cattod.2004.09.053.

[ref33] Caron S.; Dugger R. W.; Ruggeri S. G.; Ragan J. A.; Ripin D. H. B. Large-scale oxidations in the pharmaceutical industry. Chem. Rev. 2006, 106 (7), 2943–2989. 10.1021/cr040679f. 16836305

[ref34] Liang Y. F.; Jiao N. Oxygenation via C–H/C–C bond activation with molecular oxygen. Acc. Chem. Res. 2017, 50 (7), 1640–1653. 10.1021/acs.accounts.7b00108. 28636366

[ref35] Campbell A. N.; Stahl S. S. Overcoming the “oxidant problem”: strategies to use O_2_ as the oxidant in organometallic C–H oxidation reactions catalyzed by Pd (and Cu). Acc. Chem. Res. 2012, 45 (6), 851–863. 10.1021/ar2002045. 22263575 PMC3355522

[ref36] Hunsen M. Carboxylic acids from primary alcohols and aldehydes by a pyridinium chlorochromate catalyzed oxidation. Synthesis 2005, 2005 (15), 2487–2490. 10.1055/s-2005-872085.

[ref37] Jiang X.; Zhang J.; Ma S. Iron catalysis for room-temperature aerobic oxidation of alcohols to carboxylic acids. J. Am. Chem. Soc. 2016, 138 (27), 8344–8347. 10.1021/jacs.6b03948. 27304226

[ref38] Liu M.; Wang H.; Zeng H.; Li C. J. Silver (I) as a widely applicable, homogeneous catalyst for aerobic oxidation of aldehydes toward carboxylic acids in water—“silver mirror”: From stoichiometric to catalytic. Sci. Adv. 2015, 1 (2), e1500020 10.1126/sciadv.1500020. 26601150 PMC4643818

[ref39] Liu M.; Li C. J. Catalytic Fehling’s reaction: an efficient aerobic oxidation of aldehyde catalyzed by copper in water. Angew. Chem. 2016, 128 (36), 10964–10968. 10.1002/ange.201604847. 27505714

[ref40] Yu H.; Ru S.; Dai G.; Zhai Y.; Lin H.; Han S.; Wei Y. An efficient iron(III)-catalyzed aerobic oxidation of aldehydes in water for the green preparation of carboxylic acids. Angew. Chem., Int. Ed. 2017, 56 (14), 3867–3871. 10.1002/anie.201612225. 28252238

[ref41] Saisaha P.; Buettner L.; van der Meer M.; Hage R.; Feringa B. L.; Browne W. R.; de Boer J. W. Selective catalytic oxidation of alcohols, aldehydes, alkanes and alkenes employing manganese catalysts and hydrogen peroxide. Adv. Synth. Catal. 2013, 355 (13), 2591–2603. 10.1002/adsc.201300275.

[ref42] Yu H.; Ru S.; Zhai Y.; Dai G.; Han S.; Wei Y. An efficient aerobic oxidation protocol of aldehydes to carboxylic acids in water catalyzed by an inorganic-ligand-supported copper catalyst. ChemCatChem 2018, 10 (6), 1253–1257. 10.1002/cctc.201701599.

[ref43] Yang Z.; Luo R.; Zhu Z.; Yang X.; Tang W. Harnessing the reactivity of iridium hydrides by air: iridium-catalyzed oxidation of aldehydes to acids in water. Organometallics 2017, 36 (21), 4095–4098. 10.1021/acs.organomet.7b00634.

[ref44] Jeong D.; Kim H.; Cho J. Oxidation of aldehydes into carboxylic acids by a mononuclear manganese(III) iodosylbenzene complex through electrophilic C–H bond activation. J. Am. Chem. Soc. 2023, 145 (2), 888–897. 10.1021/jacs.2c09274. 36598425

[ref45] Zhang Y.; Liu Y.; Wang D.; Liu J.; Zhao J.; Chen L. State-of-the-art advances in the syntheses, structures, and applications of polyoxometalate-based metal-organic frameworks. Polyoxometalates 2023, 2 (1), 9140017 10.26599/POM.2022.9140017.

[ref46] Li J.; Zhang D.; Chi Y.; Hu C. Catalytic application of polyoxovanadates in the selective oxidation of organic molecules. Polyoxometalates 2022, 1 (2), 9140012 10.26599/POM.2022.9140012.

[ref47] Yu H.; Wang J.; Wu Z.; Zhao Q.; Dan D.; Han S.; Tang J.; Wei Y. Aldehydes as potential acylating reagents for oxidative esterification by inorganic ligand-supported iron catalysis. Green Chem. 2019, 21, 4550–4554. 10.1039/C9GC02053E.

[ref48] Xu J.; Zhang Y.; Yue X.; Huo J.; Xiong D.; Zhang P. Selective oxidation of alkenes to carbonyls under mild conditions. Green Chem. 2021, 23, 5549–5555. 10.1039/D1GC01364E.

[ref49] Zeng K.; Stückl A. C.; Qin J.; Simon M.; Spyra C. J.; Li J.; Meyer F.; Zhang K. Iodoarene mediated efficient aerobic oxidation of aldehydes for carboxylic acids. Mol. Catal. 2023, 537, 112919 10.1016/j.mcat.2023.112919.

[ref50] Upadhyay J.; Misra S. P.; Irusta S.; Sharma S.; Deshpande P. A. Oxidation of aldehydes to carboxylic acids over geopolymer supported CuO. Mol. Catal. 2023, 536, 112911 10.1016/j.mcat.2022.112911.

[ref51] Kalimuthu P.; Hege D.; Winiarska A.; Gemmecker Y.; Szaleniec M.; Heider J.; Bernhardt P. V. Electrocatalytic aldehyde oxidation by a tungsten dependent aldehyde oxidoreductase from Aromatoleum aromaticum. Chem. - Eur. J. 2023, 29 (20), e202203072 10.1002/chem.202203072. 36648073

[ref52] Lin J. C.; Yi Y.; Zhang L.; Cang R.; Ji X. J.; Zhang Z. G. A solvent-tolerant whole-cell biocatalyst for chemoselective oxidation of aldehydes to carboxylic acids. Mol. Catal. 2023, 550, 113576 10.1016/j.mcat.2023.113576.

[ref53] Vanoye L.; Favre-Réguillon A. Selective aerobic oxidation of aliphatic aldehydes: the critical role of percarboxylate anion on the selectivity. React. Chem. Eng. 2023, 8 (5), 1043–1050. 10.1039/D2RE00471B.

[ref54] Xu J.; Yue X.; He L.; Shen J.; Ouyang Y.; Liang C.; Li W. Photoinduced protocol for aerobic oxidation of aldehydes to carboxylic acids under mild conditions. ACS Sustainable Chem. Eng. 2022, 10 (43), 14119–14125. 10.1021/acssuschemeng.1c06755.

[ref55] Nakahara K.; T sriwong K.; Hawari M. A.; Tanaka A.; Matsuda T. Enzyme immobilization on a 3D-printed reactor for aldehyde oxidation to carboxylic acid under mild conditions. React. Chem. Eng. 2023, 8 (3), 543–547. 10.1039/D2RE00547F.

[ref56] Machinaga N.; Kibayashi C. 1,5-dihydro-3H-2,4-benzodioxepine as a novel carbonyl protecting group. Tetrahedron Lett. 1989, 30 (31), 4165–4168. 10.1016/S0040-4039(00)99349-3.

[ref57] Kawashima M.; Sato T.; Fujisawa T. A facile method for synthesis of three carbon-homologated carboxylic acid by regioselective ring-opening of β-propiolactones with organocopper reagents. Tetrahedron 1989, 45 (2), 403–412. 10.1016/0040-4020(89)80068-7.

[ref58] Robles J. L.; Bochet C. G. Photochemical release of aldehydes from α-acetoxy nitroveratryl ethers. Org. Lett. 2005, 7 (16), 3545–3547. 10.1021/ol051280w. 16048338

[ref59] Nunez M. T.; Martin V. S. Efficient oxidation of phenyl groups to carboxylic acids with ruthenium tetraoxide. A simple synthesis of (*R*)-gamma-caprolactone, the pheromone of *Trogoderma granarium*. J. Org. Chem. 1990, 55 (6), 1928–1932. 10.1021/jo00293a044.

[ref60] Chiang P. C.; Bode J. W. On the role of CO_2_ in NHC-catalyzed oxidation of aldehydes. Org. Lett. 2011, 13 (9), 2422–2425. 10.1021/ol2006538. 21486084 PMC3100891

[ref61] Newkome G. R.; Robinson J. M.; Sauer J. D. Pyrolysis of 2-bis(methylthio)methylpyridine S-oxides. Synthesis of substituted pyridinecarbaldehydes. J. Chem. Soc., Chem. Commun. 1974, (10), 410–411. 10.1039/c39740000410.

[ref62] Lewandowski W.; Świderski G.; Świslocka R.; Wojtulewski S.; Koczoń P. Spectroscopic (Raman, FT-IR and NMR) and theoretical study of alkali metal picolinates. J. Phys. Org. Chem. 2005, 18 (9), 918–928. 10.1002/poc.918.

[ref63] Bresch S.; Wandell R.; Wang H.; Alabugin I.; Locke B. R. Oxidized derivatives of n-hexane from a water/argon continuous flow electrical discharge plasma reactor. Plasma Chem. Plasma Process. 2016, 36 (2), 553–584. 10.1007/s11090-015-9686-x.

