# Supplementary material for: Ultrafast Solid-Phase Oxidation of Aldehydes to Carboxylic Acids by Atmosphseric Plasma Treatment
Source: ACS Omega. 2024 Jun 6;9(25):27269–77. doi: 10.1021/acsomega.4c01596 (PMC11209928; doi:10.1021/acsomega.4c01596)
Supplement: Supplementary file 1 — ao4c01596_si_001.pdf [file ao4c01596_si_001.pdf]

Supplementary Material

Ultrafast Solid Phase Oxidation of Aldehydes to Carboxylic Acids by Atmospheric Plasma Treatment

Bálint Árpád Ádám [1], Ádám Golcs [1,2,*], Tünde Tóth [1,3], Péter Huszthy [1]

[1] Department of Organic Chemistry and Technology, Budapest University of Technology and Economics, Szent Gellért tér 4., H-1111 Budapest, Hungary

[2] Department of Pharmaceutical Chemistry, Semmelweis University, Hőgyes Endre utca 9., H-1092 Budapest, Hungary

[3] HUN-REN Centre for Energy Research, Konkoly-Thege Miklós út 29-33., H-1121 Budapest, Hungary

* Correspondence: golcs.adam@semmelweis.hu

1. Failed test reactions
2. Determined yields in the experimental design
3. Validation of the experimental design
4. Effects of plasma treatment on the silica support

1. Failed test reactions

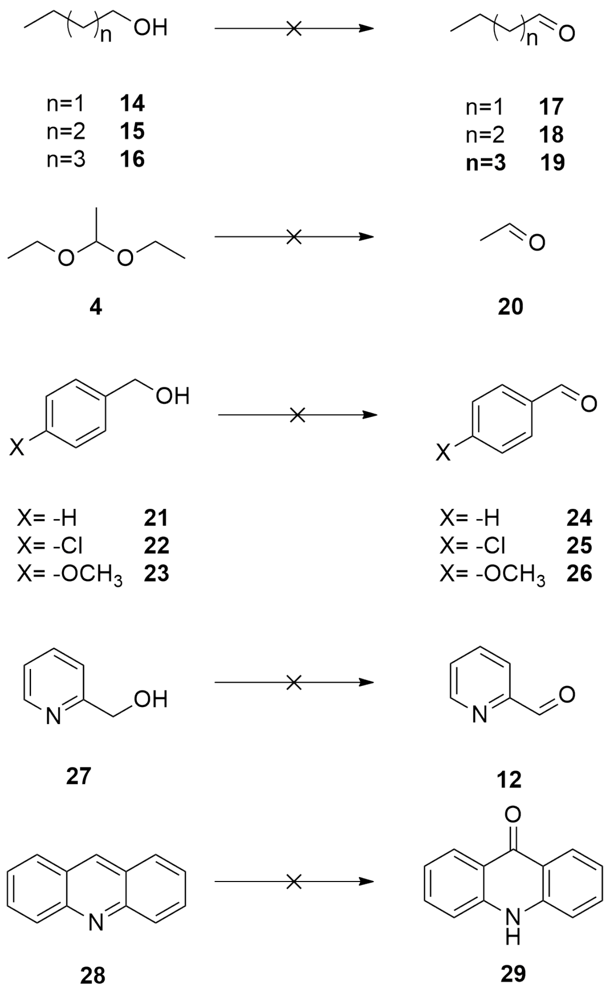

Figure S1. Failed test reactions for studying substrate scope and extendibility of the method.

2. Determined yields in the experimental design

Table S1. Results of the tests 3nt he oxidation of aliphatic model compounds

Nr.	Layer thickness (mm)	Distance (mm)	Time (s)	Yield (%)
1	0.2	10	3	100
2	0.2	10	6	100
3	0.2	10	9	100
4	0.2	20	3	80
5	0.2	20	6	100
6	0.2	20	9	100
7	0.2	30	3	0
8	0.2	30	6	5
9	0.2	30	9	15

Table S2. Results of the tests 3nt he oxidation of aromatic model compounds

Nr.	Layer thickness (mm)	Distance (mm)	Time (s)	Yield (%)
1	0.2	10	3	100
2	0.2	10	6	100
3	0.2	10	9	100
4	0.2	20	3	23
5	0.2	20	6	55
6	0.2	20	9	60
7	0.2	30	3	0
8	0.2	30	6	0
9	0.2	30	9	0

Table S3. Results of the tests 3nt he oxidation of heteroaromatic model compounds

Nr.	Layer thickness (mm)	Distance (mm)	Time (s)	Yield (%)
1	0.2	10	3	100
2	0.2	10	6	100
3	0.2	10	9	100
4	0.2	20	3	0
5	0.2	20	6	45
6	0.2	20	9	52
7	0.2	30	3	0
8	0.2	30	6	0
9	0.2	30	9	0

3. Validation of the experimental design

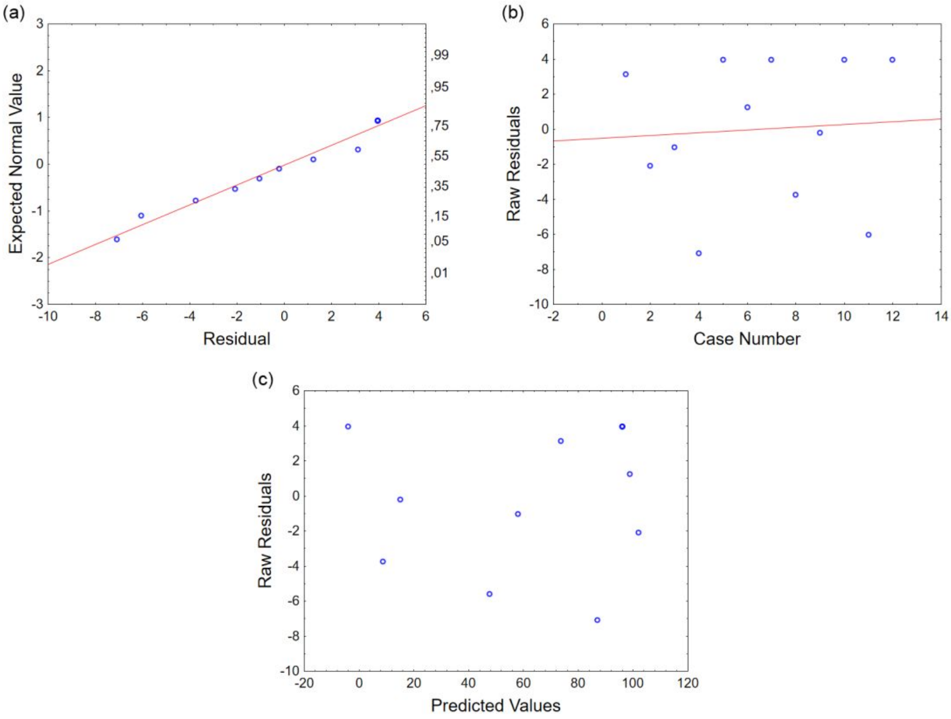

Figure S2. Statistical validation of the data analysis in the experimental design for aliphatic model compounds [(**a**): normal probability plot for checking the normality assumption; (**b**): raw residuals against case numbers for examining the independence of measurements; (**c**): predicted against residual values for proving the constancy of variances].

The analysis of a full-factorial design assumes that the observations are normally and independently distributed. To check the normality assumption a 'normal probability plot' of residuals was constructed [see part (a)]. As the residuals fall approximately along a straight line, we can conclude that the data come from a normal population. The independence of the measurements was examined with the 'Residuals vs. Case numbers' diagram on part (b). The even scattering of residues indicates that the measurements can be considered independent of each other. The constancy of variances was determined by plotting the predicted values on the x-axis and the residuals on the y-axis [see part (c)], where neither systematic arrangements nor trends were observed, i.e. the variance can be considered constant.

4. Effects of plasma treatment on the silica support

SEM measurements indicate no visible changes in morphology and porosity after plasma treatment. The average particle size of 10-12 μm (d50 laser diffraction, size distribution, according to the supplier's information) was unchanged after the plasma treatment. Melted areas were also not observed. SEM records from the untreated (see the left column) and treated samples (see the right column) in different magnifications can be seen in Figure S3.

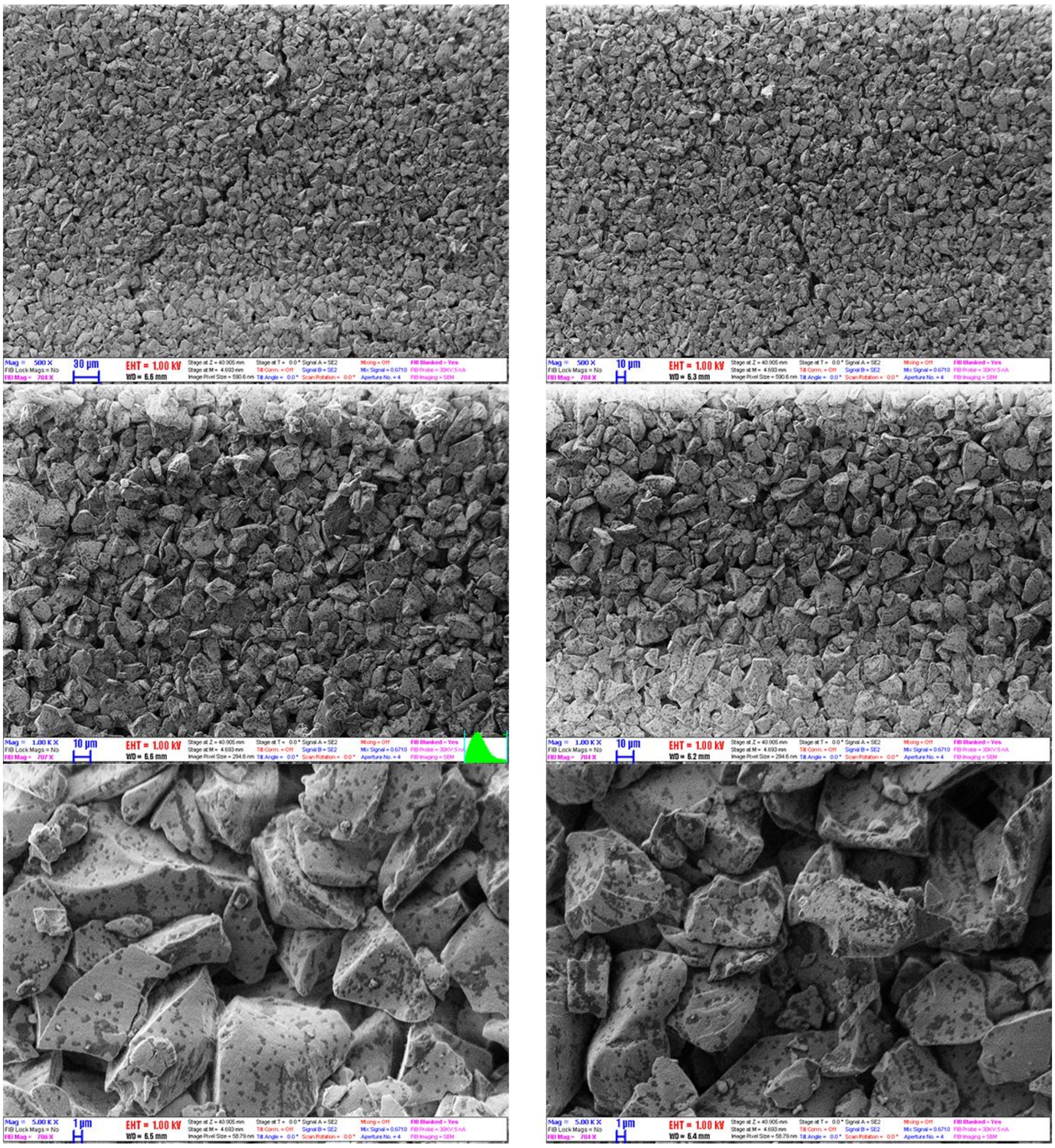

Figure S3. SEM records from the untreated (left column) and treated surface of the silica support plate (right column) in different magnifications

ATR-FTIR measurements indicate no chemical changes after plasma treatment. The ratio of the polar surface groups (e.g, Si-OH) might be increased upon the plasma treatment, the changes in their extent could not be shown spectroscopically as they were also present in the surface of the untreated support plates. Only the broad peak at 3400-3500 cm^{-1} has slightly increased, which is the *O-H* bond stretching due to the presence of adsorbed water molecules. Thus, the adsorbent was wetted by plasma. Results can be seen in Figure S4.

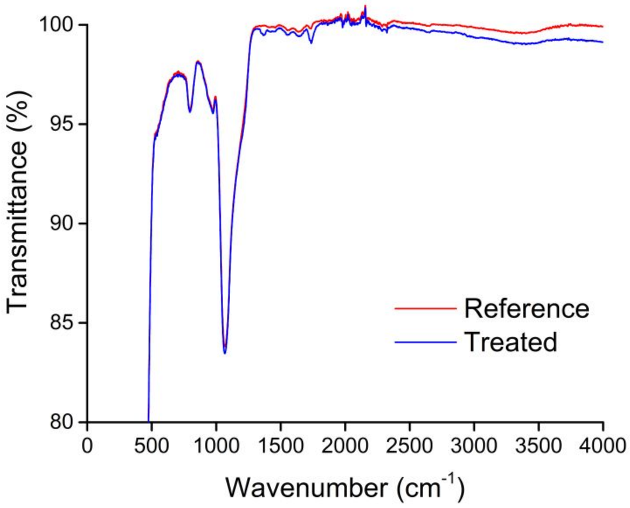

Figure S4. IR spectra of the untreated reference and the plasma treated support plates

Contact angle measurements were also performed and revealed a slightly improved wettability of the treated surface. The original surface was already a very polar one (31.5°, 7.5 μL volume of water droplet), but the contact angles were further decreased (10.0°, 7.5 μL volume of water droplet) after the plasma treatment. Probably it is due to the enhanced ratio of the end-chain Si-OH groups.

The experiments were repeated by using pretreated support plates (previously treated with atmospheric plasma beam on the whole surface under the corresponding optimized condition). The same results were obtained. Based on these results, we can conclude that under the applied conditions, the plasma treatment only has a negligible effect (slight wetting and increasing the number of end-chain Si-OH groups) on the silica support, which can be considered chemically indifferent in the oxidation process.
